# A Decision Tree for Rapid Quality Assurance and Control of Rifampicin-Containing Oral Dosage Forms for Global Distribution for Tuberculosis Treatment

**DOI:** 10.4103/0250-474X.40323

**Published:** 2008

**Authors:** Y. Ashokraj, Shrutidevi Agrawal, R. Panchagnula

**Affiliations:** Department of Pharmaceutics, National Institute of Pharmaceutical Education and Research (NIPER), Sector 67, SAS Nagar, Punjab - 160 062, India

**Keywords:** Global tuberculosis drug facility, directly observed treatment short-course, DOTS, antituberculosis drugs, rifampicin, fixed dose combinations

## Abstract

For centuries tuberculosis remained as a complex socioeconomic problem impeding human development. Directly observed treatment short-course and fixed dose combinations were implemented in tuberculosis therapy for maximum success of treatment. However, drug shortages primarily hindered the expansion of directly observed treatment short-course, which lead to development of the global tuberculosis drug facility. Since large geographical area is covered by the global tuberculosis drug facility for global drug supply for tuberculosis eradication programs, a rapid quality control and assurance has become necessary to ensure the quality and performance of supplied antituberculosis drugs. In this manuscript a decision tree is proposed for facilitating rapid quality control (*in vitro* and *in vivo*) of antituberculosis formulations procured by the global tuberculosis drug facility. This decision tree also predicted to be applicable at every stages of anti tuberculosis drug product development, especially in identification of poor quality products and monitoring batch-to-batch variability. Further, it provides opportunity for effective quality control in resource poor settings and the gained knowledge is anticipated to be applicable for development and evaluation of antimalarial and antiAIDS fixed dose combinations.

Tuberculosis (TB) remains as a challenge to mankind for centuries, causing incredible burden and persistently impeding the human development even after acquisition of powerful drugs, which assures complete cure. Though an impressive progress was evidenced in the treatment after the introduction of antibiotics, a surge of new cases of infection with drug resistance along with human immunodeficiency virus epidemic emerged as a potential threat for eradication of TB. Considering the severity and spread of disease, World Health Organization (WHO) declared TB as a global emergency, a global initiative for controlling TB (Anonymous, 1994) and implemented Directly Observed Treatment Short-course (DOTS) and use of Fixed Dose Combinations (FDCs) in worldwide TB eradication programs which significantly improved TB treatment. DOTS strategy is considered as essential for achieving the global target for TB control, which consists of detecting at least 70% of estimated infectious cases and curing at least 85% of them. Achieving this would substantially decrease the prevalence of TB, and corroborate a decrease in incidence towards the eradication. It is projected that the set target may not be reached due to low case detection and drug shortages, which are frequent and serious in many parts of the world and often limited by financial constraint, inefficient drug procurement system and poor management. Though shortage of drugs is not the unique problem of TB therapy but the consequences are extremely serious. Hence, continuous supply of drugs is considered as one of the important aspect of DOTS expansion and subsequent successful TB treatment^1^. Recognizing the urgent need and to overcome the serious constraint, Global TB drug Facility (GDF) was, developed and established by Global partnership to Stop TB program^2^. Accordingly new international approaches towards ensuring universal access and effective national system of procurement and distribution of TB drugs is devised and implemented. As a result, antiTB drugs are procured and distributed through the GDF, which promotes standardized treatment requirement and products approved by WHO for effective TB control. Since large geographical area is covered under GDF for rapid DOTS expansion, procurement was facilitated by standardized prequalification program which is approval of manufacturing firm for regular supply of antiTB drugs based on the document review, good manufacturing practice, inspection and quality control. However, quality assurance of those supplied formulations or circulating in the market is necessary to ensure the batch-to-batch uniformity after the testing of bio-batch. As the volume of products handled by GDF is very high, there is a need for rapid methodology for evaluation of drugs supplied for global distribution to TB eradication programs.

On the other hand, the quality of FDCs, a reduced bioavailability of rifampicin in certain formulations compared to loose combinations is a major impediment in wide spread use of FDCs in TB therapy. Though there were several speculations, this quality issue appears to be attributed to multiple and critical factors that affect the pharmaceutic and biopharmaceutic processes for an immediate release delivery system^3^. From the biopharmaceutic perspective, rifampicin was classified as class II drug of biopharmaceutic classification system (BCS) clearly indicating that only the factors affecting *in vivo* dissolution alters its bioavailability^4^. As this problem is not confined only to FDCs but extends to formulations containing only rifampicin also, manifests that the contribution of pharmaceutic factors are predominant. Thus, lack of suitable evaluatory test(s) e.g., proper dissolution methodology, to identify those pharmaceutic factors affecting *in vivo* dissolution and bioavailability of rifampicin is reasoned for the failure of number of biostudies. Moreover, unavailability of discriminative evaluatory method lead to failure of monitoring the quality of subsequent batches of products after biobatches^5^. Addressing this issue a dissolution testing method was proposed and validated, according to which a two medium dissolution in 0.01 N hydrochloric acid and phosphate buffer (PB) pH 6.8 at 50 rpm in United States Pharmacopoeia (USP) apparatus II would forecast the quality of formulations. A ‘multiple point’ dissolution method was recommended in order to monitor the consistency in the performance of the formulations in addition to the ‘Q’ value of ‘80% dissolution in 30 min^6^. These dissolution specifications were validated through the *in vitro-in vivo* correlations, which altogether lead to an efficient and discriminative *in vitro* methodology for quality control of rifampicin containing formulations at any stage of product cycle.

As per the model protocol published by WHO for *in vivo* evaluation of FDCs by conducting bioequivalence trials for anti-TB FDCs, 20-22 volunteers must be used with six sampling points^7^ and other local regulatory authorities (e.g., Indian drug regulatory authorities) recommend 12-14 volunteers and sampling point up to 24 h. The number of sampling points substantially increases, if the bioequivalence to be established simultaneously for other drugs (isoniazid, pyrazinamide and ethambutol) also. Thus, the whole process of bioequivalence study becomes expensive and time consuming. In order to simplify the *in vivo* evaluation, various investigations were initiated to throughput these bioequivalence studies, with respect to sample size, sample time and bio-analysis. After extensive investigation, as a modification in the existing WHO protocol a reduced sample size of 12 volunteer and plasma pooling as method of analysis was recommended^8^,^9^. These specifications not only simplify the *in vivo* evaluation but also provided opportunity for quality assurance of FDCs in resource poor settings, where the bioequivalence trials are less affordable. As a significant improvement in these biostudies, feasibility of development of FDCs as reference formulation was demonstrated^10^.

Thus, the basic understanding on the performance of rifampicin containing formulations and developed methodologies for its evaluation (both *in vitro* and *in vivo*) emerged as a decision tree for rapid and continuous evaluation and monitoring of quality of anti-TB drug products (fig. 1), which is particularly recommended for the quality evaluation of FDCs procured by various global bodies such as WHO (GDF) and Medicins Sans Frotieres, for the global TB eradication programs. This method provides rapid assessment of the quality of the products for mass distribution and advantageous than the currently recommended/adopted statutory methods (Table 1).

**Fig. 1:**
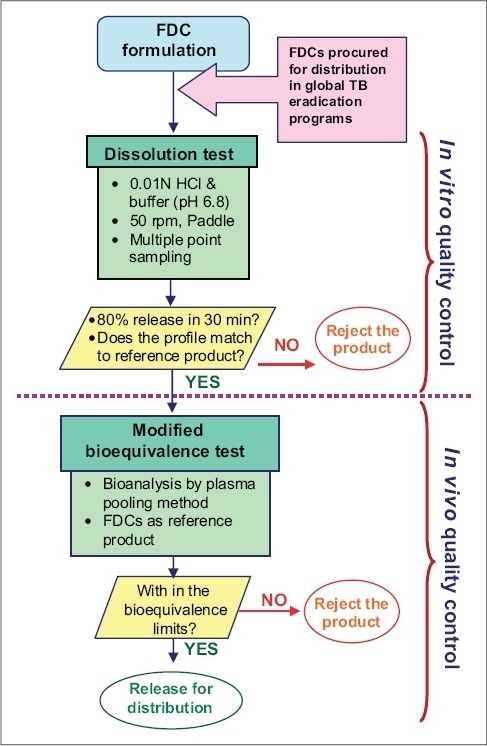
Flow chart depicting the decision making in producing and quality control of antiTB FDC products at various stages of product cycle. This decision tree is particularly recommended for the quality evaluation of FDCs which are procured by various global bodies like World Health Organization (Global drug facility for anti-TB drugs), Medicins Sans Frotieres, etc for the global TB eradication programs. It provides rapid evaluation of quality and performance of FDCs which all together saves ample time, labour and cost. The scope of this decision tree extends to whole product cycle of FDCs from providing guidance in product development to post marketing surveillance. The use of currently recommended dissolution method and bioequivalence method is practically effective in quality control of FDCs in resource poor settings. This flow chart may be extended for the evaluation of antimalarial and antiAIDS FDCs.

**TABLE 1:** SUMMARY AND COMPARISON OF STATUTORY QUALITY CONTROL SPECIFICATIONS WITH THE CURRENTLY RECOMMENDED SPECIFICATIONS WITH RESPECT TO *IN VITRO* AND *IN VIVO* EVALUATION OF RIFAMPICIN CONTAINING FDCS

QC specifications	Statutory requirements^1^	Current recommended^2^	Comments
***In vitro* dissolution**			
Media	0.1 N HCl, 0.01 N HCl, SGF with out pepsin, PB (10 mM, pH 6.8)	0.01 N HCl and PB (pH 6.8)	Only two media for all type of FDCs
Apparatus	Basket and paddle	Paddle	Avoids confusion regarding type of equipment
Agitation intensity	100 rpm	50 rpm	50 rpm is more discriminative than 100 rpm
‘Q’ value	75% dissolution in 45 min and 80% dissolution in 30 min	80% dissolution in 30 min	Assure availability of only good quality formulations
Time of sampling	Single point sampling	Multiple point sampling	Enables control of batch-to-batch to variability
***In vivo* bioequivalence test**			
Number of volunteers	22-24 (WHO protocol)	12 volunteers	Reduced cost of study
Method of analysis	Drug analysis for individual subject (WHO protocol)	Plasma pooling	Practically reduces total time for analysis from months to days
Reference product	Separate combinations	FDCs	Avoids confusion in selection of reference formulation for bioequivalence trials

1 Statutory requirements include pharmacopoeial specifications (USP) for dissolution test and details in the model protocol published by WHO for bioequivalence trials.

2 Current indicates the specifications for dissolution as well as bioequivalence studies that can be applied at any stage of product cycle of FDCs, which includes during development, routine QC of manufacturing batches and post marketing surveillance. QC is quality control; SGF stands for simulated gastric fluid and PB for phosphate buffer

As shown in fig. 1, antiTB FDCs procured/supplied need to be subjected for dissolution testing using 0.01 N HCl and PB at 50 rpm in USP apparatus II, with multiple point sampling method. If 80% dissolution in 30 min was achieved in both the media and the dissolution profiles match with the reference formulation, the product should be subjected further to bioequivalence test. In bioequivalence test with a minimum number of volunteers and using FDCs as reference product a biostudy need to be conducted by the truncated model protocol provided by WHO. Further, the plasma samples should be analyzed by plasma pooling method and the bioequivalence assessed using appropriate statistical analysis. If the product passes the test it can be allowed for distribution. Otherwise the product should be rejected at the stages given in the decision tree.

The unique advantage with this decision tree, especially for evaluation of antiTB drug products supplied for mass distribution, is its rapidity in producing the results. The recommended plasma pooling methodology virtually saves ample amount of time from months to days. Further, the procured product will be utilized as soon as possible and wastage of drugs due to expiration before usage could be avoided. In addition, the volume of products, which could be evaluated by this decision tree substantially increases, thus, facilitating complete quality check of all the products supplied for global distribution. For the evaluation of different set of batches of same product the *in vivo* bioequivalence test may be waived and restricted only for dissolution testing, which further facilitates the rapid distribution of products. Overall, it provides expedition on evaluation of quality and performance of FDCs which all together saves enormous time, labor and cost in the quality assurance.

The scope of this decision tree not only applies with the continuous monitoring of drug products supplied for GDF but also applicable at any stage of the product cycle. The recommended dissolution method is predicted to forecast the exact *in vivo* performance of the rifampicin containing products, thus, efficiently identifies good and poor quality products. Another potential issue to be controlled is batch-to-batch variability in the performance of not only FDC products but also in rifampicin alone products. The present dissolution method provides opportunity to monitor batch-to-batch quality through the multiple point dissolution method. Further, this method alone could be utilized for quality evaluation of drug products in resource poor setting where bioequivalence testing is not feasible or affordable. In addition, identification of appropriate reference formulation for bioequivalence studies of generic versions is facilitated by the present method of evaluation. The present knowledge may be extended and applied for the evaluation of antimalarial and antiAIDS FDCs also.

In a nut shell, the developed decision tree for quality assurance and control of antiTB products have the following salient features; i. efficient identification of only good quality formulations, ii. isolate ‘false negative’ as well as ‘false positive’ FDCs, iii. assure bath-to-batch consistency with respect to quality and performance, iv. facilitate the reduction of development time and simplify registration process, v. enable effective quality control in resource poor setting and vi. identification of appropriate reference formulation for bioequivalence studies
